# Supplementary material for: 25-Hydroxycholesterol Induces Intrinsic Apoptosis via Mitochondrial Pathway in BE(2)-C Human Neuroblastoma Cells
Source: Int J Mol Sci. 2025 Aug 19;26(16):8012. doi: 10.3390/ijms26168012 (PMC12386542; doi:10.3390/ijms26168012)
Supplement: Supplementary file 1 [file ijms-26-08012-s001.zip › ijms-3817156-supplementary.pdf]

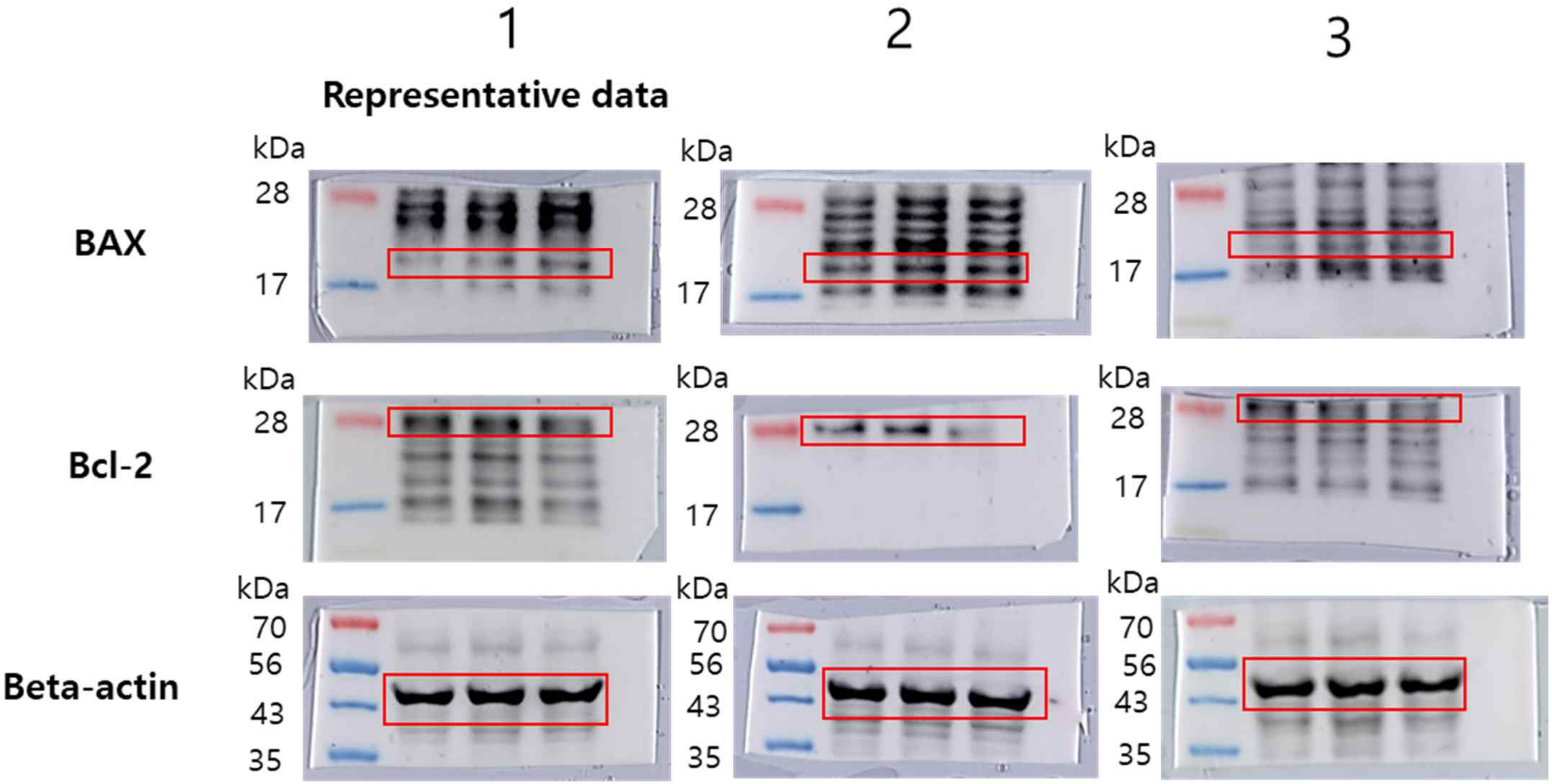

1
2
3
Representative data
BAX
Bcl-2
Beta-actin
kDa
28
17
kDa
28
17
kDa
28
17
kDa
28
17
kDa
28
17
kDa
28
17
kDa
70
56
43
35
kDa
70
56
43
35
kDa
70
56
43
35

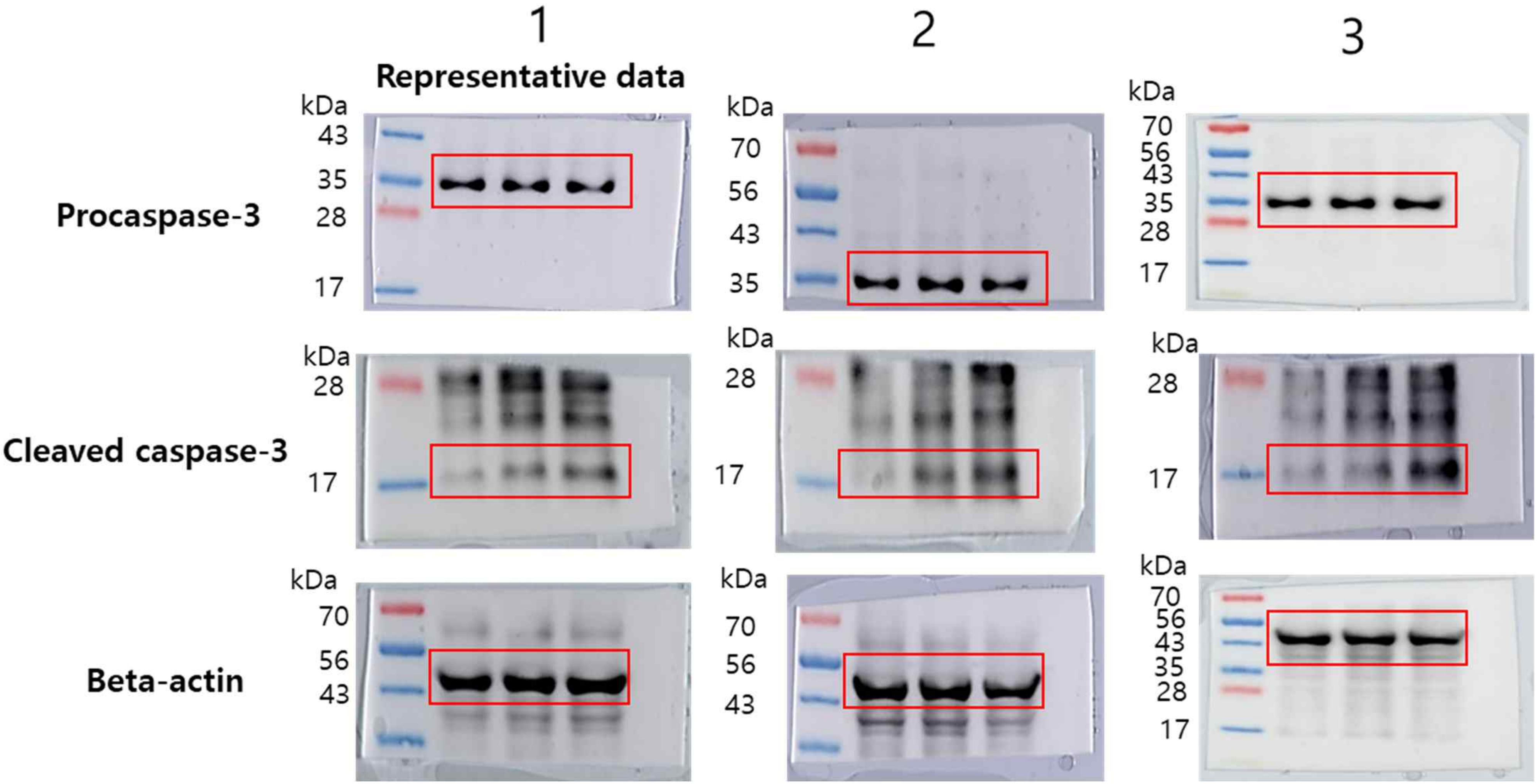
1
Representative data
2
3
Procaspase-3
Cleaved caspase-3
Beta-actin
kDa
43
35
28
17
70
56
